# Multipoint Pacing versus conventional ICD in Patients with a Narrow QRS complex (MPP Narrow QRS trial): study protocol for a pilot randomized controlled trial

**DOI:** 10.1186/s13063-016-1698-1

**Published:** 2016-12-03

**Authors:** Maurizio Gasparini, Paola Galimberti, Renato Bragato, Stefano Ghio, Claudia Raineri, Maurizio Landolina, Enrico Chieffo, Maurizio Lunati, Ederina Mulargia, Alessandro Proclemer, Domenico Facchin, Roberto Rordorf, Alessandro Vicentini, Lina Marcantoni, Francesco Zanon, Catherine Klersy

**Affiliations:** 1 Humanitas Research Hospital, Via Manzoni 56, Rozzano, Italy; 2 Fondazione IRCCS Policlinico S. Matteo, Pavia, Italy; 3 Ospedale Maggiore, Crema, CR Italy; 4 AO Niguarda Cà Granda, Milano, Italy; 5 Ospedale S. Maria della Misericordia, Udine, Italy; 6 Ospedale Santa Maria della Misericordia, Rovigo, Italy; 7 Servizio di Biometria e Statistica, Fondazione IRCCS Policlinico S. Matteo, Pavia, Italy

**Keywords:** Narrow QRS, Multipoint pacing, Cardiac resynchronization therapy, ICD, Heart failure

## Abstract

**Background:**

Despite an intensive search for predictors of the response to cardiac resynchronization therapy (CRT), the QRS duration remains the simplest and most robust predictor of a positive response. QRS duration of ≥ 130 ms is considered to be a prerequisite for CRT; however, some studies have shown that CRT may also be effective in heart failure (HF) patients with a narrow QRS (<130 ms). Since CRT can now be performed by pacing the left ventricle from multiple vectors via a single quadripolar lead, it is possible that multipoint pacing (MPP) might be effective in HF patients with a narrow QRS. This article reports the design of the MPP Narrow QRS trial, a prospective, randomized, multicenter, controlled feasibility study to investigate the efficacy of MPP using two LV pacing vectors in patients with a narrow QRS complex (100–130 ms).

**Methods:**

Fifty patients with a standard ICD indication will be enrolled and randomized (1:1) to either an MPP group or a Standard ICD group. All patients will undergo a low-dose dobutamine stress echo test and only those with contractile reserve will be included in the study and randomized. The primary endpoint will be the percentage of patients in each group that have reverse remodeling at 12 months, defined as a reduction in left ventricular end-systolic volume (LVESV) of >15% from the baseline.

**Discussion:**

This feasibility study will determine whether MPP improves reverse remodeling, as compared with standard ICD, in HF patients who have a narrow QRS complex (100–130 ms).

**Trial registration:**

ClinicalTrials.gov, NCT02402816. Registered on 25 March 2015.

**Electronic supplementary material:**

The online version of this article (doi:10.1186/s13063-016-1698-1) contains supplementary material, which is available to authorized users.

## Background

There is considerable evidence that cardiac resynchronization therapy (CRT) has beneficial effects in heart failure (HF) patients [1, 2]. According to current guidelines, CRT is not recommended if QRS duration is < 130 ms [3]. However, as many as 59% of HF patients with reduced ejection fraction (EF) have a QRS duration of < 120 ms [4]. Although some studies have reported a positive effect of CRT in HF patients with a narrow QRS (<120 ms) [5–7], others have reported that CRT has no benefit in these patients [8]. Recently, a technique for pacing from multiple points in the left ventricle (LV) via a single quadripolar lead has been introduced (multipoint pacing [MPP]). Preliminary studies have demonstrated an acute improvement in LV dP/dt_Max_ [9] and hemodynamic parameters based on pressure-volume loops [10]. Furthermore, MPP has been shown to shorten the QRS duration in comparison with conventional biventricular pacing [11], shorten LV activation time [12], and improve both medium- and long-term outcomes [13, 14]. Pacing from multiple LV sites may improve the response to CRT by capturing a larger area of myocardial tissue, improving depolarization and repolarization patterns, and capturing areas adjacent to scar tissue [15].

Aranda et al. reported that the mean QRS variability may be > 30 ms in patients with HF and a QRS duration ≤ 130 ms [16]. These wide fluctuations in QRS duration over time may be problematic, moving some patients in and out of the CRT indication window. Thus, there may be a “gray zone” of patients with a QRS between 100 and 130 ms who could benefit from CRT. Furthermore, one of the main difficulties in CRT studies in patients with a narrow QRS is that biventricular stimulation in these patients usually results in a QRS that is longer than the baseline QRS. This QRS prolongation may partially offset the potential benefit of CRT in HF patients with a narrow QRS [5].

These patients, with LVEF ≤ 35 and narrow QRS, still have an indication for ICD implantation.

Incidentally, from the MADIT-CRT study results, despite implantable cardioverter defibrillators (ICDs) preventing sudden cardiac death, it does not say that an improvement is obtained in term of ventricular remodeling [17].

In this randomized, prospective, multicenter feasibility trial, we will evaluate the potential benefit of MPP via a quadripolar lead in HF patients with a narrow QRS (100–130 ms) and a standard indication for implantation of an ICD. Patients with no left ventricular contractile reserve, as determined by low-dose dobutamine stress echocardiography test, will be excluded from the study. The control group will consist of patients who receive an ICD only without any CRT. The results of this pilot study could provide important information on the potential benefits of MPP in patients with a narrow QRS complex.

## Methods

### Study population and randomization

This study is a prospective, randomized, multicenter, physician-initiated, pilot study. This is a parallel and superiority study. The study will be conducted at several Italian centers (up to nine high-volume Italian hospitals). Enrollment will begin in 2016 and is expected to continue through the beginning of 2017, until the predefined target number of patients has been reached. The expected duration of the investigation will be approximately 2–3 years, with 24 months of follow-up for each patient. Prior to enrollment, approximately 70–80 patients will be screened by means of an echo-dobutamine stress (DSE) test in order to evaluate the presence of left ventricular contractile reserve. Low-dose dobutamine stress echocardiography (LDSE) is a simple, cost-effective, and widely available method of identifying contractile reserve in the LV [18, 19]. The dose of intravenous dobutamine will be increased in 5 μg kg^−1^ min^−1^ increments every 5 minutes up to a recommended maximum dose of 20 μg kg^−1^ min^−1^. The test will be terminated in the event of arrhythmias or the occurrence of any complications or side effects. Measurements (offline) of end-diastolic and end-systolic volumes will be assessed at each infusion rate by two-dimensional echocardiography (biplane disk method). The left ventricular ejection fraction (LVEF), measured offline by means of Simpson’s biplane quantitative method, will be determined at each infusion rate (including baseline). An absolute increase of 5 points or higher in LVEF above the baseline will demonstrate the presence of LV contractile reserve and will be considered a positive LDSE test [18]. Only patients who have a positive LDSE test will be enrolled if they meet the inclusion criteria reported in Table 1.

**Table 1:** Inclusion/exclusion criteria

A. Inclusion criteria
● HF with NYHA class II/III ● LVEF < 35% in patients on OPT for at least 3 months and candidate for ICD ● Patients with left ventricular contractile reserve at low-dose dobutamine stress echocardiography test
● QRS duration: 100–130 ms
● Ability and willingness to comply with study requirements
● Successful quadripolar LV lead implantation (only for treatment group)
B. Exclusion criteria
● Myocardial infarction, unstable angina within 40 days prior to enrollment
● Cardiac revascularization (PTCA, stent or CABG) in the 4 weeks prior to enrollment or planned for the 3 months following enrollment
● Cerebrovascular accident (CVA) or transient ischemic attack (TIA) in the 3 months prior to enrollment
● Primary valvular disease
● Inability to comply with the follow-up schedule
● Age less than 18 years
● Pregnancy or intention to become pregnant during the period of the investigation
● Classification of status 1 for cardiac transplantation or consideration for transplantation over the next 12 months
● Previous cardiac transplantation
● Life expectancy < 12 months
● Permanent atrial fibrillation

*HF* heart failure, *NYHA* New York Heart Association, *LVEF* left ventricular ejection fraction, *OPT* optimized therapy, *ICD* implantable cardioverter defibrillator, *LV* left ventricle, *PTCA* percutaneous transluminal coronary angioplasty, *CABG* coronary artery bypass graft surgery

Patients who meet the eligibility criteria and do not have any exclusion will be recruited by the clinical cardiology groups associated with each enrolling center. Logs will be kept at each center of all identified patients who meet the clinical eligibility criteria. For eligible patients who are not enrolled, the reason for non-enrollment (exclusion) will be recorded.

The enrolled patients will be randomized in a 1:1 fashion to an MPP group or a Standard ICD group (control group).

Randomization will be centralized and stratified by center in order to obtain a balanced distribution of the devices implanted at each center. The random assignment to one of the two study groups will be made by the clinical research organization (CRO) and transmitted to the enrolling clinical center by logging on to a web-based automated program.

**Fig. 1:**
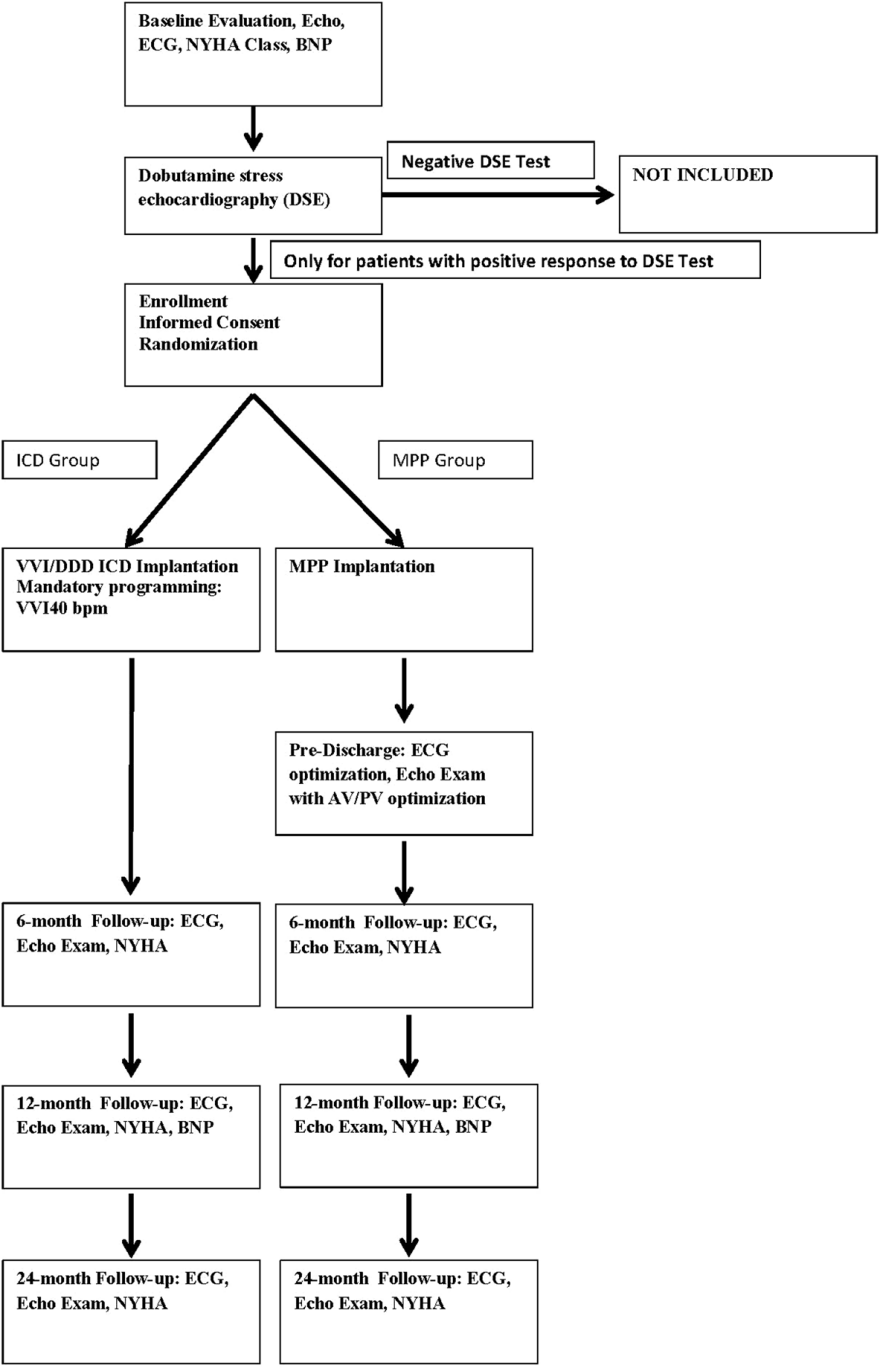
Study flow chart of the MPP Narrow QRS trial. Data will be collected from enrollment to 24-month follow-up

A flow diagram of patients in the study and a list of all investigational specific activities/procedures (Additional file 1) are shown in Fig. 1 and Table 2.

**Table 2:** List of all investigational-specific activities/procedures

	Time point	Enrollment	Implant	Pre-discharge	6-12-24 month follow-up	Implant	6-12-24 month follow-up
	Study activity	ALL patients		MPP group		Standard ICD group	
Enrollment	Low-dose dobutamine stress echocardiography	X					
	Informed consent process	X					
	Verification of inclusion/exclusion criteria	X					
	Demographics and medical history	X					
	Randomization	X					
Intervention:	MPP device implant		X				
	Standard ICD implant					X	
Assessment	Fluoro images and/or cine of venogram and final lead position, two projections		X			X	
	Capture thresholds and signal amplitude measurements on RA and RV leads		X	X	X	X	X
	Capture thresholds and phrenic nerve stimulation occurrence at LV pacing configurations		X	X	X		
	NYHA functional class	X			X		X
	QRS duration	X		X	X		X
	Standard echo measurements	X			X		X
	Electrical delay measurements			X	X		
	QRS optimization			X	X		
	12-lead ECG during QRS optimization			X	X		
	Echo measurements and AV optimization			X	X		
	Optimal programming of the device			X	X		

*MPP* multipoint pacing, *ICD* implantable cardioverter defibrillator, *RA* right atrium, *RV* right ventricle, *LV* left ventricle *NYHA* New York Heart Association, *ECG* electrocardiogram, *AV* atrioventricular

All the enrolled patients must be indicated for ICD implantation in accordance with the current guidelines. [3] (An ICD is recommended in order to reduce the risk of sudden death and all-cause mortality in patients with symptomatic HF (NYHA Class II–III) and an LVEF ≤ 35% despite ≥ 3 months of OMT, provided they are expected to survive substantially longer than 1 year with good functional status).

### Implantation procedure

The implantation procedure will be carried out within 30 days of enrollment. All subjects will receive either a market-approved St. Jude Medical (Saint Paul, MI, USA) CRT device that includes the MPP feature (models numbers CD3271-40(Q), CD3371-40(Q), CD3371-40C (QC) or newer) and a St. Jude Medical quadripolar left ventricular lead (Quartet 1458Q or newer) or a market-approved St. Jude Medical ICD device. Any commercially available and CE-marked right atrial and right ventricular leads may be implanted. A Merlin® Patient Care System (model 3650 or newer) programmer will be used to interrogate and program all devices on implantation and during follow-up. Post-implantation fluoroscopic images in two views will be required to document lead locations. Before hospital discharge, patients with an MPP device will undergo A-V delay optimization by means of the Ritter method, in order to maximize diastolic filling [20]. During this session, two MPP pacing vectors will be selected from the quadripolar lead. The pacing vectors will be selected to minimize interventricular conduction delay. Following selection of the two MPP vectors, V-V timing optimization will be performed according to the optimal pacing intervention identified by means of QRS duration criteria to avoid lengthening of the QRS duration. In the ICD group, every physician can decide to implant a ventricular (VVI) or traditional dual-chamber (DDD) ICD, but programming must be VVI 40 bpm [21, 22]. In case of LV lead implant failure the patient will be considered a dropout on treatment analysis.

### Study measurements and endpoints

All patients enrolled will undergo a baseline examination for collection of the following information: medical history and clinical evaluation, assessment of QRS duration, assessment of New York Heart Association (NYHA) functional class, echocardiographic examination, including evaluation of LV volumes and LVEF, mitral regurgitation grade, and inter- and intraventricular dyssynchrony estimation [23]. Plasma brain natriuretic peptide (BNP) levels will also be collected at the baseline and all follow-up visits. A complete echocardiographic analysis will be performed at the baseline and follow-up visits. The echo data will be analyzed by a central echo core laboratory, which will be blinded to allocation.

All data will be collected on an online database created for this study. Every effort will be made to ensure compliance with this schedule.

If a visit is missed for any reason, data on endpoints and adverse events will be collected at the subsequent visit.

All deaths will be reported immediately to the coordinator center.

### Primary endpoint

The primary endpoint is the percentage of patients who display reverse remodeling in the MPP group, in comparison with the ICD group, at the 12-month follow-up examination. Reverse remodeling is defined as a decrease in LVESV > 15% from the baseline [20].

### Secondary endpoints

The secondary endpoints are:

1.  the percentage of patients presenting with reverse remodeling in the MPP group compared with the Standard ICD group at the 24-month follow-up examination;
2.  the percentage of patients in each group who have an absolute increase of > 5 points in LVEF at the 12- and 24-month follow-up examinations;
3.  the percentage of patients with a BNP value < 400 pg/mL at the 12-month follow-up compared between groups.

Echocardiographic images will be acquired by the echocardiography laboratory at each participating center. Pavia Hospital (Fondazione IRCCS San Matteo, Pavia, Italy) will serve as the blind central core laboratory for the analysis of all echocardiographic results.

### Protection of human subjects

At each clinical center, the routine follow-up interval history, physical examination, and device interrogation will be used to identify possible adverse events. Each physician is responsible for ensuring that the therapies rendered are consistent with the well-being of the patients.

If a situation arises where it is in the best interest of the patient that the programming be changed, the device will be programmed according to patient's need.

All information and data collected for the MPP Narrow QRS trial concerning subjects or their participation in this investigation will be considered confidential by all parties involved in the trial.

### Data monitoring and quality control

Study data will be monitored closely by the CRO and coordinator center (CC). Periodic reports will be generated on data completion and error rates for each clinical center.

In addition, all study data will undergo an extensive computer edit, and this information will be provided to the clinical centers to help improve and maintain data quality control procedures designed to detect inaccuracies and inconsistencies.

This information will be used to make decisions about relevant adjustment procedures in the study procedures.

All data are managed and maintained by the CC and the CRO.

### Rationale for the choice of a one-sided 90% confidence interval (CI)

For our pilot trial, we wish to identify a sample size that is large enough to meet the following requirements: (a) if the observed difference between the two groups in the pilot trial is zero, then the upper confidence limit will exclude the estimate that is considered “clinically significant” in the planned definitive trial; (b) reasonable confidence that our pilot trial is big enough to enable us to make the right decision on whether or not to proceed to a larger trial [24]. A 90% confidence interval would provide reasonable certainty regarding trial decision-making, but would be small enough to deliver a study within a reasonable budget and time frame. Furthermore, we propose to use a one-sided CI, as we are only interested in proceeding toward the main trial if there is some evidence of effectiveness. If the intervention appeared to be harmful, even if this effect were not statistically significant, we would not proceed.

### Sample size calculation

A main trial designed to show an effect size of +23% - from a proportion of success of 17% to an expected proportion of success of 40% - with alpha = 5% and power = 90% would require us to enroll 79 patients per group (total 158 patients). In the pilot study, recruiting, randomizing and analyzing 50 participants (25 per group), assuming that 17% approximately four patients) would respond in each group, would produce a 13% upper limit of the one-sided 90% confidence interval; this would exclude us finding a 23% difference, which would be statistically significant in a larger trial. In the event of 10% attrition (23 patients per group enrolled. i.e., incomplete data set due to deviation from protocol, implant failure or missing follow-up) the upper limit of the one-sided 90% confidence interval will be extended to 14.5%. If attrition is higher than 10%, a corresponding increase in sample size will be considered, in order to obtain 45–50 evaluable patients.

Finally, it has been estimated [18] that 30% of the screened patients will have a negative LDSE test. Therefore, about 70–80 patients will be screened for participation in the study, 70% of whom are likely to be enrolled and randomized in the study.

### Statistical analysis and decision-making process

The intervention effect will be reported as the difference in the proportions of success and 90% CI. No formal test of hypothesis will be undertaken, as we are only interested in whether the hypothesized treatment estimate is larger than 0. If in this pilot study with that sample size we find an estimate larger than zero, and the upper limit of the one-sided 90% confidence interval excludes the clinically relevant effect size of 23% under the null hypothesis, and the pilot also shows that it were feasible to recruit and retain the participants, and the intervention is not harmful, then the recommendation would be to move forward with the main study. Re-evaluation of the effect size to be used for sample size computation in the main study will take into account the results from the pilot study.

## Discussion

Major clinical trials in CRT have used prolonged QRS duration as one of the key inclusion criteria [1, 25]. Current European Society of Cardiology (ESC) guidelines do not recommend CRT if QRS duration is < 130 ms [3]. However, 59% of patients with an EF < 35% have a QRS duration of < 120 ms according to Brignole et al. [4]. Previous studies on CRT in patients with a narrow QRS complex have shown discordant results. Some preliminary studies have reported positive results in patients with a narrow QRS complex [5–7]. Gasparini et al. [26] and Achilli et al. [6] reported that CRT had both clinical and functional benefits that were similar in patients with either a wide or narrow QRS. Yu et al. evaluated CRT in 102 HF patients with a narrow QRS and coexisting mechanical dyssynchrony assessed by tissue Doppler imaging [7]. They found that CRT resulted in LV reverse remodeling and an improved clinical status. In a prospective study with 36 months of follow-up, we reported a significant reduction of end-systolic volume (from 127.4 ± 29.7 mL to 55.6 ± 23.5 mL) in 45 patients with a QRS < 120 ms [27]. Bleeker et al. reported comparable results, with an improvement in LVEF and a reduction in LV end-systolic volume in patients with a narrow QRS (<120 ms) [28]. In the Narrow CRT study, Muto et al. demonstrated that 46% of patients with a narrow QRS had an improved clinical composite score after 1 year of follow-up [29]. In contrast to these studies, both RethinQ [30] and the Esteem study [31] reported that CRT in patients with a QRS < 120 ms did not significantly increase the proportion of patients with improvement in peak oxygen consumption (VO_2_). However, a subgroup of patients in RethinQ with a QRS of 120–130 ms displayed an improvement in peak VO_2_ on CRT. The LESSER-EARTH study [32] found that CRT did not improve clinical outcomes or left ventricular remodeling. Moreover, the Echo CRT study, which enrolled patients with systolic heart failure and a QRS duration < 130 ms, found that CRT did not reduce the rate of death or hospitalization [8]. Since the publication of this study, the CRT guidelines have been changed [3].

Multipoint pacing is a novel pacing technology and might increase the number of patients that respond to CRT. Pacing from multiple LV sites may improve the response of these patients by capturing a larger area of myocardial tissue, improving depolarization and repolarization patterns, and capturing areas adjacent to scar tissue. Studies of activation patterns reveal that MPP is able to recruit a greater portion of the LV, generating a flat wave-front with a higher conduction velocity [12]. Zanon et al. showed that MPP induced contractility improvements, measured by dP/dt, in association with QRS narrowing, when compared with conventional biventricular pacing [11]. Pappone et al. showed that the acute and long-term results of CRT in HF patients were similar, regardless of whether the QRS was < 150 ms or > 150 ms [13, 14]. The first studies on this new technology seem to underline [11–14] an increase in responders and a reduction in QRS duration in MPP patients.

Molhoek et al. [33] demonstrated that baseline QRS was not predictive of response: however, responders showed a significant reduction in QRS duration directly after initiation of CRT, and this was maintained during follow-up.

These assumptions have led us to hypothesize that non-responder patients to a classic CRT (narrow QRS) may positively respond to a MPP pacing.

To date, no investigation has been conducted to study the effect of MPP in HF patients with a baseline QRS between 100 and 130 ms. The MPP Narrow QRS trial will enroll only patients with a standard indication for an ICD, and with left ventricular contractile reserve at DSE and a QRS between 100 and 130 ms. In these patients, who are on optimal pharmacological therapy and have an ICD indication, MPP could have an important therapeutic benefit in comparison with conventional ICD therapy alone. Furthermore, MPP will be optimized by using electrocardiogram (ECG) criteria, in order to avoid QRS lengthening. The results of this important feasibility study could provide additional scientific information on the beneficial effects of MPP in a subgroup of HF patients who are not currently indicated to receive CRT. The results could be used for a larger trial evaluating also hard endpoints.

### Trial status section

The trial is ongoing, currently four patients have been enrolled in the study.

## Additional file

Additional file 1: Standard Protocol Items: Recommendations for Interventional Trials (SPIRIT) 2013 checklist. (DOC 120 kb)
